# The Effect of Calcipotriol on the Expression of Human *β* Defensin-2 and LL-37 in Cultured Human Keratinocytes

**DOI:** 10.1155/2009/645898

**Published:** 2010-02-22

**Authors:** Beom Joon Kim, Yong Kwan Rho, Hye In Lee, Mi Sook Jeong, Kapsok Li, Seong Jun Seo, Myeung Nam Kim, Chang Kwun Hong

**Affiliations:** ^1^Department of Dermatology, Chung-Ang Medical Research Center, College of Medicine, Chung-Ang University, Seoul 156-755, South Korea; ^2^Chung-Ang Medical Research Center, College of Medicine, Chung-Ang University, Seoul 156-755, South Korea

## Abstract

*Background*. Vitamin D has been reported to regulate innate immunity by controlling the expression of antimicrobial peptides (AMPs). *Objective*. We investigated the effect of calcipotriol on the expression of AMPs in human cultured keratinocytes. *Methods*. Keratinocytes were treated with lipopolysaccharide (LPS), TNF-*α*, Calcipotriol and irradiated with UVB, cultured, and harvested. To assess the expression of human beta defensin-2 and LL-37 in the control group, not exposed to any stimulants, the experimental group was treated with LPS, TNF-*α*, or UVB, and another group was treated again with calcipotriol; reverse transcriptase-polymerase chain reaction, Western blotting, and immunohistochemical staining were performed. *Results*. In the experimental group treated with LPS, UVB irradiation, and TNF-*α*, the expression of *β*-defensin and LL-37 was increased more than in the control group and then decreased in the experimental group treated with calcipotriol. *Conclusions*. Calcipotriol suppressed HBD-2 and LL-37, which were stimulated by UVB, LPS, and TNF-*α*.

## 1. Introduction

Antimicrobial peptides (AMPs) are small molecular weight proteins with a broad spectrum of antimicrobial activity against bacteria, viruses, and fungi [1, 2]. They are involved in the first line of defense as well as coordination of the innate and adaptive immune system [3]. In addition, recent studies have shown that AMPs can function as chemokines, proteinases, and neuropeptides [4]. Many AMPs have been found in a variety of tissues such as respiratory, urogenital, and skin epithelium [5–7]. Human ß-defensin (HBD) and cathelicidin have been identified as the principal peptides in the skin. 

These AMPs exhibit an imbalance or dysregulation in some inflammatory skin diseases such as atopic dermatitis and psoriasis. LL-37 as a peptide form of human cathelicidin and HBD-2 both have been reported to be downregulated in the skin of patients with atopic dermatitis compared to patients with psoriasis [6]. Unlike atopic dermatitis, HBD and cathelicidin are strongly increased in keratinocytes in psoriatic plaques [8]. Considering these findings, AMPs have been thought to play a role in the pathogenesis of inflammatory skin diseases. 

The mechanisms underlying the regulation of AMP expression remain poorly understood. Many cytokines, Toll-like receptors (TLRs), and vitamin D are thought to be involved in the regulation of AMP expression. The activation of Toll-like receptors has been reported to lead to the nuclear factor kappa B (NF*κ* *B*) dependent induction of a variety of AMPs including the defensins [9–11]. HBD-2 and -3 have been reported to be induced by interleukin (IL)-1*α*, IL-*β*, and the tumor necrosis factor (TNF)-*α* [10, 12]. 

Recently, vitamin D has been reported to be important in cutaneous immune modulation as well as calcium regulation and bone metabolism. Vitamin D induces epidermal keratinocytes to growth arrest, differentiation, and changes in cytokine expression [13]. In addition, vitamin D has been associated with an increase in the expression of LL-37 in cultured human keratinocytes and in human skin in vivo [14]. Moreover, the vitamin D response element has been identified in the cathelicidin promoter [15]. Vitamin D-induced cathelicidin worsens the inflammation associated with psoriasis; however, vitamin D has been used for the treatment of psoriasis. The molecular effects of vitamin D on the AMPs have not been elucidated in patients with psoriasis. 

In this study, we investigated the effects of calcipotriol, a vitamin D_3_ analogue on the expression of AMPs in human cultured keratinocytes. The results showed that calcipotriol suppressed the upregulated expression of hBD-2 and LL-37, stimulated by ultraviolet B (UVB), lipopolysaccharide (LPS), and TNF-*α*.

## 2. Materials and Methods

### 2.1. Cell Culture

 The keratinocytes were cultured in Keratinocyte Basal Medium (Cambrex, Walkersville, MD., USA), supplemented with KGM SingleQuots (Cambrex, Walkersville, MD., USA) grown in a 75 cm^2^ flask and incubated with 5% CO2 at 37°C. The cultured keratinocytes were divided in the amount of 2 × 10^5^/mL and plated in a standard flat bottomed 10 cm^2^ polystyrene plate. The cells were starved overnight in KBM supplemented with free KGM. Some cells were then irradiated with 20 mJ/cm^2^ of UVB and treated with LPS (Sigma, St. Louis, MO., USA) 5 *μ*g/mL, TNF-*α* (Sigma, St. Louis, MO., USA) 100 U/mL, and Calcipotriol (Sigma, St. Louis, MO., USA) 10^−9^ *μ*g and incubated for 6, 12, and 24 hours.

### 2.2. Ultraviolet B Irradiation (UVB)

 The dose of irradiation used was 20 mJ/cm^2^; this dose was chosen based on preliminary data. UVB irradiation was delivered with a Philips TL 20 W/12 (Eindhoven, The Netherlands), a fluorescent bulb emitting 280–320 nm wavelength with a peak at 313 nm. Before UVB irradiation, the medium was removed and covered with phosphate buffered saline (PBS). Irradiation output was monitored with a Waldmann UV-meter (Waldmann, Villigen-Schwenningen, Germany).

### 2.3. Preparation of Primers

We synthesized the PCR primers based on Gene Bank data. The primers were chemically synthesized using a DNA synthesizer (Pharmacia, Björkgatan, Uppsala, Sweden). The sequences were as follows:

**hBD- (128 bp):**

5′-ATC TCC TCT TCT CGT TCC TC-3′ (sense),

5′-ACC TTCTAG GGC AAA AGA CT-3 (anti-sense).

**LL- (203 bp):**

5′-CTG ATG CCT CTT CCA GGT GT-3′ (sense),

5′-GAG GGA GCC CTT TCT GAA TC-3′ (anti-sense).

**GAPDH (593 bp):**

5′-CCA CCC ATG GCA AAT TCC ATG GCA-3′ (sense),

5′-GGT GCT GCT TGT TAG GAG GTC AAG TAA AGG GC-3′ (anti-sense).

### 2.4. Reverse Transcription-Polymerase Chain Reaction (RT-PCR)

Total RNA was isolated from the cultured keratinocytes using TRIzol reagent (Invitrogen, Carlsbad, CA, USA). To the cells, 1 mL of TRIzol reagent was added in a culture dish. After 5 minutes at room temperature, 0.2 mL of chloroform was added per 1 mL of TRIzol reagent; the tubes were shaken vigorously by hand for 15 seconds and then they were incubated at 15°C to 30°C for 3 minutes. The mixtures were centrifuged at 12,000 rpm at 4°C for 15 minutes, the upper aqueous phase was transferred to a fresh tube, and the same volume of 2-propanol was added. After the incubation at 4°C for 15 minutes, the specimen was centrifuged at 12,000 rpm at 4°C for 15 minutes. The supernatant was removed, then washed with 500 *μ*l of 70% ethanol, and centrifuged at 12,000 rpm at 4°C for 5 minutes; the RNA pellet was then briefly dried. The purified RNA was dissolved in diethyl pyrocarbonate- distilled water (DEPC-DW) 30 *μ*l. Next, 3 *μ*g of total cellular RNA was reverse transcripted at 42°C for 30 minutes in 20 *μ*l volume containing 1 *μ*l reverse transcriptase (TaKaRa, Shiga, Japan), 10X buffer 2 *μ*l, 10 mM dNTP 2 *μ*l (dNTP mix), oligo dT primer 1 *μ*l, RNase inhibitor 0.5 *μ*l, and 25 mM MgCl2 4 *μ*l. 

Then, 2 *μ*l of each cDNA sample from the RT-PCR was amplified by PCR in 25 *μ*l containing 10X buffer 2.5 *μ*l, 25 mM MgCl2 2.5 *μ*l, and 10 pmol 0.75 *μ*l primer. 

The thermal cycle profiles were as follows: 94°C for 5 minutes, 35 cycles at 94°C for 1 minute, 59°C for 1 minute, 72°C for 1 minute, and a final extension step at 72°C for 10 minutes.

### 2.5. Electrophoresis

The products were run on 1.5% agarose gel containing 1 *μ*g of ethidium bromide per millimeter; 20 *μ*l of reaction mixture was mixed with loading buffer separated by electrophoresis for 15 minutes at 100 voltage and visualized by UV transillumination.

### 2.6. Quantitative Analysis

Densitometry was performed on the hybrids of the PCR products of hBD-2, LL-37 and GAPDH, and the DIG on chemiluminescent film was calculated (volume of hBD-2/volume of GAPDH X100, volume of LL-37 /volume of GAPDH X100).

### 2.7. Statistical Analysis

The amount of hBD-2 and LL-37 in the keratinocytes between the control and stimulated groups was statistically compared using the ANOVA.

### 2.8. Western Blotting

The cultured keratinocytes were lysed in a buffer containing 50 mM Tris-Cl (pH 8.0), 150 mM NaCl, 0.02% sodium azide, 100 *μ*g/mL phenylmethanesulfonyl fluoride (PMSF), 1 *μ*g/mL aprotinin, and 1% Triton X100, and then centrifuged at 12,000 rpm at 4°C for 30 minutes. The supernatant was transferred to a new tube. Then, 30 *μ*g of soluble protein was loaded onto a 15% sodium dodecyl sulfate polyacrylamide gel for electrophoresis (SDS-PAGE) with a sample buffer containing 1 M Tris and glycerol 50%; the samples were heated at 95°C for 5 minutes prior to loading the gel. 

For detection of LL-37 and hBD-2, the separated proteins, from the gel electrophoresis, were transferred onto a nitocellulose membrane (Osmonics, Milwaukee, WI, USA) at 0.16 A for 1 hour. The membrane was washed three times with Tris-buffered saline tween 20 (TBST) and blocked with 5% skim milk for 1 hour at room temperature. Following this, the membrane was incubated overnight at 4°C with goat antihuman hBD-2 polyclonal antibodies (1 : 1000 in 5% bovine serum albumin, SantaCruz, Delaware, CA, USA) or goat antihuman LL-37 polyclonalantibodies (1 : 1000 in 5% bovineserum albumin, SantaCruz, Delaware, CA, USA) and then washed three times with TBST. Then, the secondary mouse anti-goat peroxidase conjugated antibodies (1 : 2000 in blocking solution, SantaCruz, Delaware, CA, USA) were incubated for 1 hour at room temperature. 

After washing the membrane with TBST, the membrane was developed with electrochemiluminescence (ECL) solution (SantaCruz, Delaware, CA, USA) for 3 minutes then exposed to X-ray film (Roche, Indianapolis, IN, USA).

### 2.9. Immunohistochemistry (IHC) for hBD-2

The keratinocytes were cultured on a coverslip (Nunc, Rochester, NY, USA). The cells were fixed for 10 minutes in 4% paraformaldehyde and washed three times for 5 minutes with PBS. Endogenous peroxidase was inactivated by incubation with 3% hydrogen peroxide at room temperature for 5 minutes and blocked with 3% BSA for 20 minutes followed by washing. The cells were incubated overnight at 4°C with goat anti-hBD-2 polyclonal antibodies, diluted 1 : 100 in PBS, then rinsed three times with PBS, and incubated with donkey antigoat peroxidase-conjugated antibodies (1 : 200 in PBS) for 1 hour at room temperature. After washing three times, the cells were immersed in 3,3′-diaminobenzidine (DAKO, Glostrup, Denmark) and then rinsed with distilled water.

### 2.10. Immunohistochemistry (IHC) for LL-37

The keratinocytes were cultured on a coverslip (Nunc, Rochester, NY, USA). The cells were fixed for 10 minutes in 4% paraformaldehyde and washed three times for 5 minutes with PBS. Endogenous peroxidase was inactivated by incubation with 3% hydrogen peroxide at room temperature for 5 minutes and blocked with 3% BSA for 20 minutes, followed by washing. The cells were incubated overnight at 4°C with goat anti-LL-37 polyclonal antibodies, diluted 1 : 100 in PBS, rinsed three times with PBS, and incubated with donkey antigoat peroxidase-conjugated antibodies (1 : 200 in PBS) for 1 hour at room temperature. After washing three times, the cells were immersed in 3,3′-diaminobenzidine (DAKO, Glostrup, Denmark) and rinsed with distilled water.

## 3. Results

### 3.1. Results for hBD

#### 3.1.1. RT-PCR

**Figure 1:**
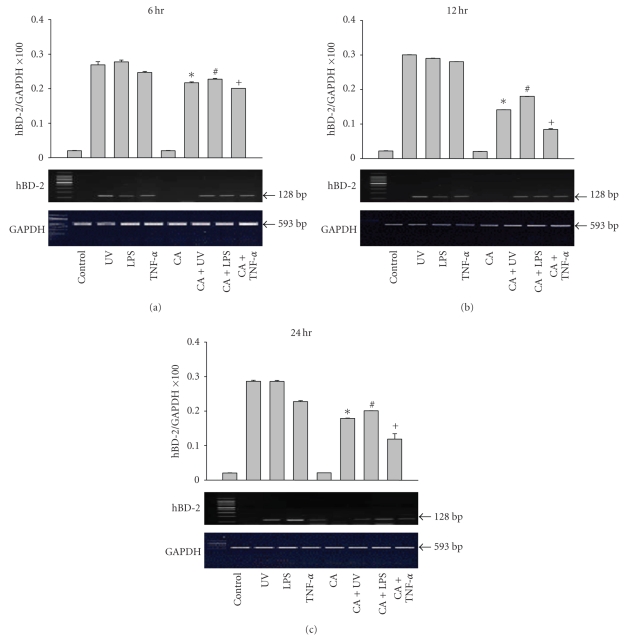
The expression of hBD-2 mRNA in keratinocytes was upregulated when stimulated with LPS, TNF-*α*, and UVB irradiation at 6, 12, and 24 hours. The hBD-2 mRNA expression was decreased after treatment with calcipotriol (CA). ∗: Statistically significant between UV and CA + UV (*P* < .01). #: Statistically significant between LPS and CA + LPS (*P* < .01). +: Statistically significant between TNF-s and CA + TNF-a (*P* < 0.01).

hBD-2 mRNA expression was not detected in the cultured keratinocytes of the unstimulated controls. The HBD-2 mRNA expression was upregulated in the UVB-irradiated, LPS, and TNF-*α*-stimulated groups. The stimulant-induced upregulation of HBD-2 mRNA expression was decreased after treatment with calcipotriol (Figure 1). These results were statistically significant when compared to the results of the control group (*P* < .001).

#### 3.1.2. Western Blotting

**Figure 2:**
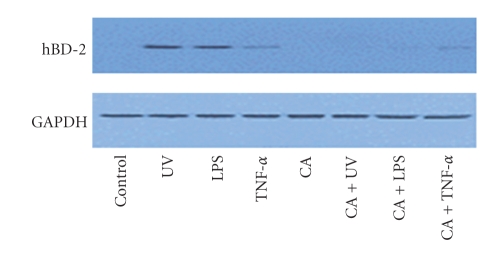
Expression of hBD protein in keratinocytes were evaluated by Western blotting using polyclonal antibody to hBD at 6, 12, and 24 hours post stimulation. The expression of hBD was decreased after additional treatment with calcipotriol compared to stimulation with LPS, TNF-*α*, and UVB irradiation in the keratinocytes.

hBD-2 protein expression was assessed by Western blotting using polyclonal antibodies against hBD-2 at 6, 12, and 24 hours after stimulation. The levels of hBD-2 protein expression in the UVB-irradiated, LPS, and TNF-*α*-treated group were more intense than in the unstimulated control group. The expression of hBD decreased after additional treatment with calcipotriol compared to the stimulated group, with LPS, TNF-*α*, and UVB irradiation in the keratinocytes (Figure 2).

#### 3.1.3. Immunohistochemistry (IHC)

**Figure 3:**
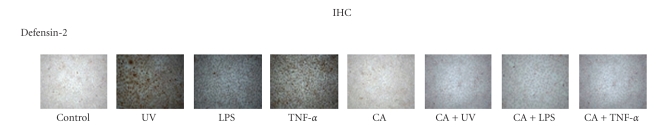
Immunostaining for hBD was more intense on LPS, TNF-*α* treated, and UV irradiated groups than in the normal control. In the additional groups treated with calcipotriol for hBD, the results were less intense than the stimulant groups.

hBD-2 protein expression in the keratinocytes was quantified via IHC analysis. The UVB-irradiated, LPS, and TNF-*α*-stimulated groups stained more strongly for hBD-2 protein expression than did the unstimulated groups. In the additional groups treated with calcipotriol, there was a less intense response than with exposure to the stimulants (Figure 3).

### 3.2. Results of LL-37

#### 3.2.1. RT-PCR

**Figure 4:**
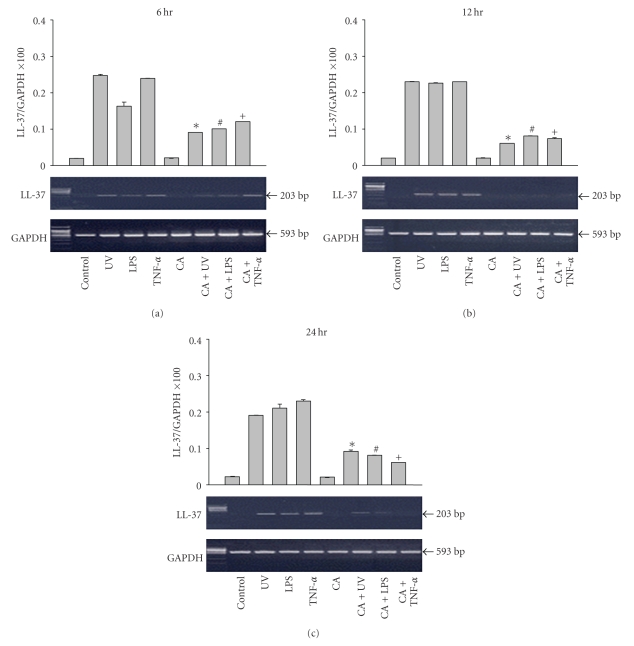
The expression of LL-37 mRNA in the keratinocytes was also upregulated when stimulated with LPS, TNF-*α*, and UVB irradiation at 6, 12, and 24 hours. Similar to the hBD-2 results, LL-37 mRNA expression decreased after treatment with calcipotriol. ∗: Statistically significant between UV and CA + UV (*P* < .01). #: Statistically significant between LPS and CA + LPS (*P* < .01). +: Statistically significant between TNF-s and CA + TNF-a (*P* < .01).

The expression of LL-37 mRNA in keratinocytes was also upregulated when stimulated with LPS, TNF-*α*, and UVB irradiation at 6, 12, and 24 hours. Similar to the results of hBD-2, LL-37 mRNA expression was decreased after treatment with calcipotriol (Figure 4). These results were statistically significant when compared to the results of the control group (*P* < .001).

#### 3.2.2. Western Blotting

**Figure 5:**
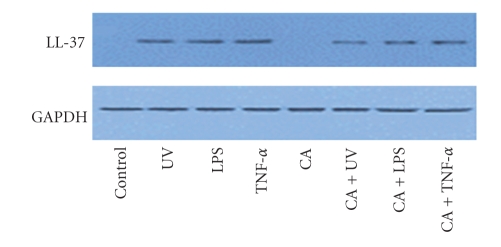
The expression of LL-37 protein in keratinocytes was evaluated by Western blotting using polyclonal antibody to LL-37 at 6, 12, and 24 hours post stimulation. The expression of LL-37 was downregulated after additional treatment with calcipotriol compared to the stimulated keratinocytes with LPS, TNF-*α*, and UVB irradiation.

LL-37 protein expression was assessed via Western blotting using polyclonal antibodies against hBD-2 at 6, 12, and 24 hours after stimulation. The level of LL-37 protein expression in the UVB-irradiated, LPS, and TNF-*α*-treated groups was more intense than observed in the unstimulated control group. The expression of LL-37 decreased after additional treatment with calcipotriol compared to the keratinocytes stimulated with LPS, TNF-*α*, and UVB irradiation (Figure 5).

#### 3.2.3. Immunohistochemistry (IHC)

**Figure 6:**
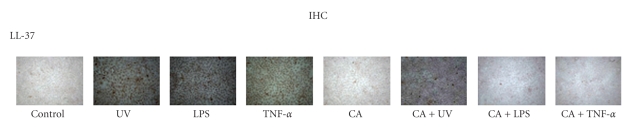
Immunostaining of LL-37 was more intense in the LPS, TNF-*α* treated, and UV irradiated groups than in the normal control. In the additional groups treated with calcipotriol, immunostaining for LL-37 was less intense than for the stimulant groups.

LL-37 protein expression in the cultured keratinocytes was quantified by IHC analysis. The UVB-irradiated, LPS and TNF-*α*-stimulated groups stained more strongly for LL-37 protein expression than did the unstimulated groups. In the additional groups treated with calcipotriol the results were less intense than when exposed to the stimulants (Figure 6).

## 4. Discussion

 AMPs were first found to act as endogenous antibiotics involved in destroying microbes. Currently, they are thought to play an important role in triggering and coordinating innate and adaptive immunity. Among the more than 20 AMPs, the cathelicidins and defensins are the best characterized in the skin. Defensins, as cationic peptides, contain 6 to 8 cysteine residues that form characteristic disulfide bridges [16]. Among alpha, beta, and theta defensins, HBDs 1 to 4 are expressed in keratinocytes. HBDs 2 to 4 can be induced by calcium and phorbol 12 myristate 13 acetate (PMA) and can be inhibited by retinoic acid [17]. Cathelicidins are an important AMP family in the skin, and the precursor protein, human cationic antimicrobial peptide 18 kDa (hCAP 18), is processed to LL-37 [18]. In human keratinocytes, cathelicidins are induced by infections, interleukin-6, and wounds [19, 20]. 

The expression and function of the AMPs are important for the appropriate modulation of immunity. In the case of atopic dermatitis, the expressions of both HBD-2 and LL-37 are significantly decreased in skin lesions [6]. These distinct defects of immune defense account for the increased incidence of skin infections with this disorder. The reduction of AMP expression is thought to be caused by the inhibitory effects of IL-4 and IL-13 on TNF-*α* and interferon (IFN)-*γ* stimulation in keratinocytes [21]. 

The mechanisms of AMP regulation in keratinocytes are incompletely understood. The expression of the AMPs is affected by various factors such as UVB, infections, inflammatory cytokines, and vitamin D. Cathelicidin is induced by vitamin D when TLR-2 and the cytokine transforming growth factor-*β* are activated after a skin injury [20]. Low-dose UVB has been reported to upregulate the AMPs and a permeability barrier functions via vitamin D [22]. TNF-*α* induces the expression of HBD-2 and 3 [9, 12]. The results of this study confirmed prior findings that UVB, LPS, and TNF-*α* increased the expression of HBD-2 and LL-37. 

 Vitamin D is an important regulator of cutaneous immunity in addition to its role in calcium homeostasis and bone metabolism. It has been reported to regulate innate immunity and enable efficient antimicrobial defenses. Several research groups confirmed that cathelicidin expression is regulated through the vitamin D3 pathway and cathelicidin is a direct target of vitamin D3 in keratinocytes [14, 15]. In keratinocytes, human cathelicidin is directly regulated by 1,25-dihydroxyvitamin D3 (1,25D3) through a vitamin D3 responsive element (VDRE) in the human cathelicidin gene CAMP [23]. Additional elements of the vitamin D3 signalling cascade have been identified that lead to increased cathelicidin such as recruitment of coactivators or epigenetic changes such as histone acetylation [24]. This inducing effect of vitamin D analogs on cathelicidin expression was confirmed in cell culture experiments using primary keratinocytes: Vitamin D analogs such as calcipotriol induced cathelicidin through activation of the VDR and subsequent CAMP transcription.

Several studies have shown that AMPs are also involved in inflammatory skin diseases such as rosacea and psoriasis [25, 26]. Recent publications highlight the role of dysregulated expression of AMPs in the pathogenesis of psoriasis. HBD-2 and cathelicidin are strongly increased in keratinocytes in psoriatic plaques6. In a recent study, cathelicidin LL-37 isolated from lesional skin was shown to form complexes with human self-DNA to activate plasmocytoid dendritic cells (pDCs) [8]. These pDCs released IFN-*α* to activate the T cell response leading to cutaneous inflammation. In addition, cathelicidin has been shown to induce the expression of proinflammatory cytokines in keratinocytes, angiogenesis, chemotaxis of immune cells, and wound repair [27–29]. 

Strategies to decrease cathelicidin in keratinocyte by targeting vitamin D3 metabolism and signaling might be beneficial in psoriasis. However, paradoxically for a long time vitamin D3 analogs have been used in the therapy of psoriasis. As vitamin D3 analogs activate the vitamin D receptor, we would expect that they induce cathelicidin presumably aggravating inflammation in psoriasis. Still, the opposite is true: vitamin D analogs are a mainstay in the topical treatment of psoriasis. They ameliorate cutaneous inflammation and reverse morphological changes within lesional skin [30]. To date mechanisms that could explain this paradoxical effect of vitamin D analogue for psoriasis have not been completely examined yet. 

This study demonstrated that previously increased AMPs like psoriatic skin lesions could be suppressed by calcipotriol. As seen in this study with RT-PCR, Western blotting, and immunohistochemical staining, upregulated HBD-2 and LL-37 were suppressed after treatment with calcipotriol, a vitamin D analogue. Although its inhibitory action was not time-dependent, calcipotriol decreased the expression of the AMPs stimulated by UVB, LPS, and TNF-*α*. Therefore, vitamin D appears to downregulate increased HBD-2 and LL-37 in stimulated keratinocytes. Thus, we speculate that vitamin D might induce AMPs under noninflammatory conditions, on the contrary downregulate previously induced AMPs under inflammatory conditions like psoriatic skin. Recent published study analyzed the expression of HBD and cathelicidin in psoriatic plaques before and after treatment with the vitamin D analogue calcipotriol [31]. Keratinocytes in lesional psoriatic plaques showed decreased expression of the AMPs HBD2 and HBD3 after topical treatment with calcipotriol. In contrast, cathelicidin antimicrobial peptide expression was increased by calcipotriol. Also, vitamin D analogs induced cathelicidin through activation of the vitamin D receptor and MEK/ERK signaling and blocked IL-17A induced HBD2 expression in cultured human epidermal keratinocyte. These studies suggest that vitamin D analogs differentially alter AMP expression in lesional psoriatic skin and cultured keratinocytes. Although our esperimental results are contrary to current understanding of vitamin D effect on AMP, these results also might present possibility for another pathway of vitamin D analogs in psoriasis patients. Vitamin D signaling pathway is very complex and consists of various elements. Further study that could make clear the mechanism for explaining downregulating effect of vitamin D on the induced AMP expression is processing now. Future experimental work on the molecular pathways and clinical correlations are needed to understand AMP and vitamin D.

In conclusion, calcipotriol suppressed induced HBD-2 and LL-37, stimulated by UVB, LPS, and TNF-*α*. These findings suggest that vitamin D modulates the expressions of AMPs in chronic skin diseases with highly expressed AMPs.
